# Crystal structure of (±)-1-({[4-(all­yloxy)phen­yl]sulfan­yl}meth­yl)-2-(di­phenyl­thio­phosphor­yl)ferrocene

**DOI:** 10.1107/S2056989015013560

**Published:** 2015-07-29

**Authors:** Audric Michelot, Stéphanie Sarda, Jean-Claude Daran, Eric Deydier, Eric Manoury

**Affiliations:** a Université de Toulouse, UPS, INPT, F-31077 Toulouse Cedex 4, France; b CNRS, LCC (Laboratoire de Chimie de Coordination), IUT, Département de Chimie, avenue Georges Pompidou, BP258, 81104 Castres, France; c CIRIMAT, équipe PPB, Université Paul Sabatier, ENSIACET – Bureau 2-r1-5, 4 Allée Emile Monso BP 44362, 31432 Toulouse Cedex 4, France; d Laboratoire de Chimie de Coordination, 205 route de Narbonne, 31077 Toulouse, Cedex 04, France

**Keywords:** crystal structure, ferrocenyl ligands, planar chirality, asymmetric catalysis

## Abstract

The title compound, [Fe(C_5_H_5_)(C_27_H_24_OPS_2_)], is built up from a ferrocene moiety substituted in the 1- and 2-positions by {[4-(all­yloxy)phen­yl]sulfan­yl}methyl and di­phenyl­thio­phosphoryl groups, respectively. The two S atoms lie on opposite sides of the cyclo­penta­dienyl ring plane to which they are attached. In the crystal, C—H⋯S hydrogen bonds link the mol­ecules into a ribbon running parallel to the (-110) plane. C—H⋯π inter­actions link the ribbons to form a three-dimensional network.

## Abstract 2

The title compound is a ferrocene derivative substituted in 1,2 positions by a di­phenyl­thio­phosphoroyl and a {[4-(all­yloxy)phen­yl]sulfan­yl}methyl chain.

## Chemical context   

Homogenous asymmetric catalysis by transition metals has received considerable attention over the last few decades and numerous chiral ligands and complexes allowing high efficiency reactions have been reported (Jacobsen *et al.*, 1999 ▸; Börner, 2008 ▸). Amongst the various chiral ligands which have been synthesized, ferrocenyl phosphines have proven to be very efficient for numerous asymmetric reactions (Buergler *et al.*, 2012 ▸; Gómez Arrayás *et al.*, 2006 ▸; Toma *et al.*, 2014 ▸). We have long been inter­ested in the synthesis of chiral ferrocenyl ligands for asymmetric catalysis (Audin *et al.*, 2010 ▸; Bayda *et al.*, 2014 ▸; Wei *et al.*, 2012 ▸; Loxq *et al.*, 2014 ▸) and, in particular, we synthesized a series of chiral P,S-ferrocenyl ligands with planar chirality, which have been successfully used in different homogeneous asymmetric catalytic reactions, such as allylic substitution, meth­oxy­carbonyl­ation and hydrogenation (Kozinets *et al.*, 2012 ▸; Diab *et al.*, 2008 ▸). We recently started to explore the grafting of these ligands on solid support. This will allow us to work in heterogeneous conditions favoring both easy catalyst separation from products and recycling. Beside the expected catalyst activity reduction observed under heterogeneous conditions compared to homogeneous reaction, surface–catalyst inter­action has proven to play an important, and still unclear, role on selectivity. A better understanding of these inter­actions would improve both grafting inter­est and probably industrial applications of such systems.

**Figure 1:**
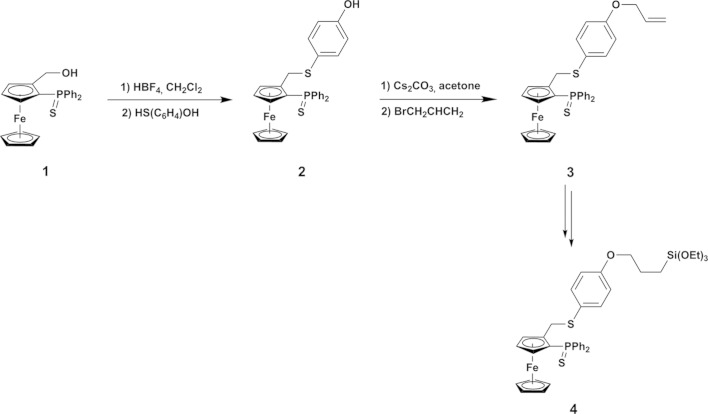
Chemical pathway showing the formation of the title compound, (**3**).

To reach this goal, we needed to developed new chiral P,S-ferrocenyl ligands bearing an alkene moiety such as compound (**3**), allowing polymerization or functionalization for inorganic grafting of the ligand [such as compound (**4**)] (Fig. 1 ▸). Functionalized P,S ferrocenyl phosphine is prepared in a three-step synthesis from 2-thiodi­phenyl­phosphino(hy­droxy­meth­yl)ferrocene (**1**) (Fig. 1 ▸). This compound can be prepared in multigram qu­anti­ties and isolated as a racemic mixture or in an enanti­omerically pure form, opening direct access to chiral ligands (Mateus *et al.*, 2006 ▸). Its functionalization can be performed in a one-pot process by successive addition of a strong acid (HBF_4_), generating probably a ferrocenyl carbocation, and then the nucleophile thiol. Addition of a base allows to generate the phenolate which reacts with bromo­allyl giving rise to compound (**3**). The phosphoryl group, protected from oxidation by sulfuration in order to carry out the former steps in air, can be recovered by refluxing in toluene with P(NMe_2_)_3_.
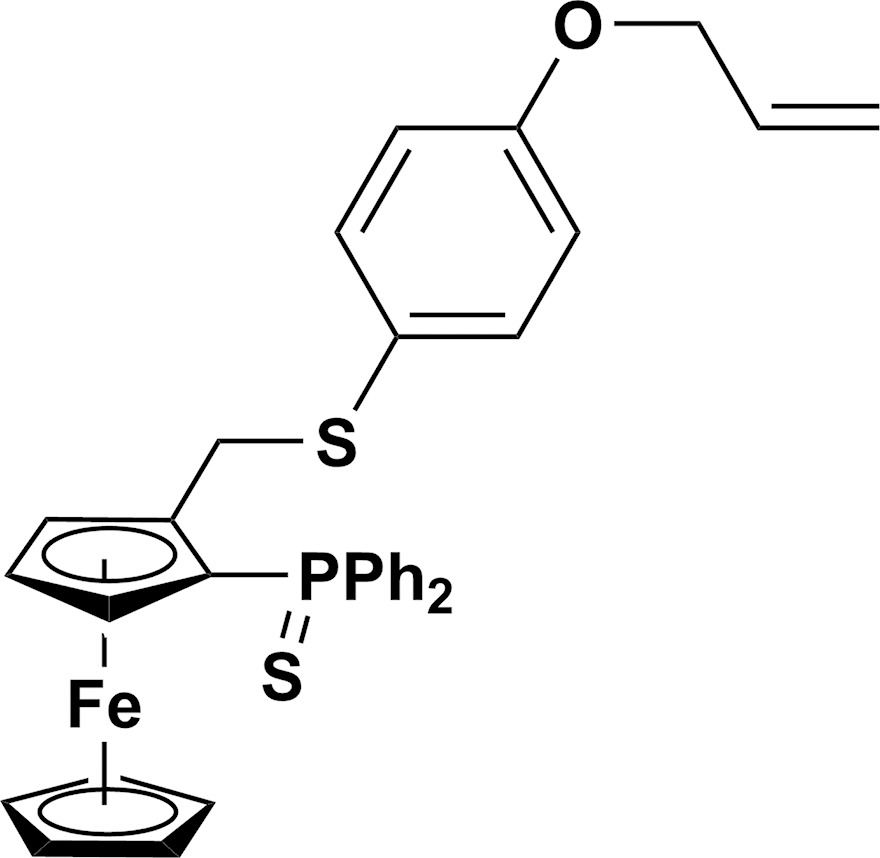


## Structural commentary   

**Figure 2:**
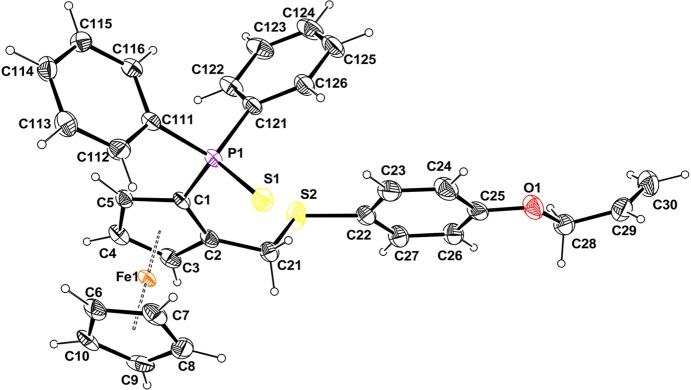
Mol­ecular view of the title compound, showing the atom-labelling scheme. Displacement ellipsoids are drawn at the 50% probability level. H atoms are represented as small spheres of arbitrary radii.

The mol­ecular structure of compound (**3**) (see Scheme) is built up from a ferrocene moiety substituted by a di­phenyl­thio­phosphoryl and a {[4-(all­yloxy)phen­yl]sulfan­yl}methyl chain (Fig. 2 ▸). As observed in other (di­phenyl­thio­phosphor­yl)ferrocenes (Table 1 ▸), the S atom (S1) of the di­phenyl­thio­phosphoryl group is *endo* towards Fe with respect to the Cp ring with a distance to the ring of 1.263 (5) Å (a perpendicular distance of S1 to the Cp ring plane). This distance is the largest one observed within similar structures. The difference observed might be related to the occurrence of the C30—H30*B*⋯S1(−*x*, −*y*, −*z*) hydrogen bond. Atom S2 is *exo*, with a distance to the Cp ring of 1.763 (4) Å, which is in agreement with the values observed for related compounds. The much shorter distance, 0.457 Å, is related to the lowest angle (15.77°) observed between the C2/C21/S2 plane and the Cp ring. In all other compounds, including the title one, the C2/C21/S2 plane is roughly perpendicular to the Cp ring, with values ranging from 71.83 to 89.50° (Table 1 ▸).

The geometry of the ferrocenyl is identical to related compounds with the two Cp rings nearly parallel to each other with a dihedral angle of 3.94 (15)° in the title compound, whereas the corresponding values range from 0.70 to 2.38° in the other compounds (Table 1 ▸). The two Cp rings are roughly eclipsed, with a twist angle of 2.8 (2)°. As observed in Table 1 ▸, the geometry of the C—PSPh_2_ and C—CH_2_-S fragments are roughly identical within experimental error. In the di­phenyl­thio­phosphoryl group, the C1—P1 distances range from 1.788 (4) to 1.802 (3) Å, whereas the P1—S1 distances range from 1.956 (2) to 1.961 (1) Å. In the C—CH2-S fragment, the C2—C21 distances range from 1.488 (2) to 1.502 (11) Å, whereas the C21—S2 distances range from 1.811 (3) to 1.835 (2) Å.

## Supra­molecular features   

**Figure 3:**
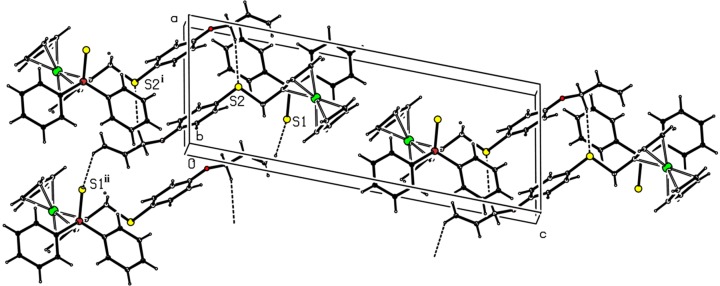
Packing view in projection down the *b* axis, showing the C—H⋯S hydrogen bonds (dashed lines). H atoms are represented as small spheres of arbitrary radii. [Symmetry codes: (i) −*x* + 1, −*y* + 1, −*z*; (ii) −*x*, −*y*, −*z*.]

**Figure 4:**
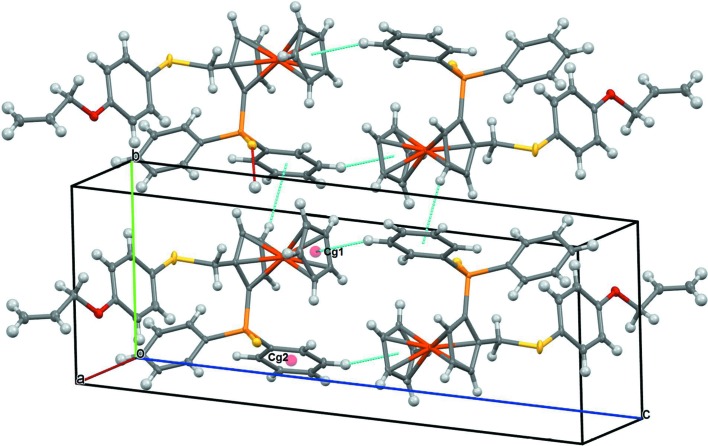
*Mercury* (Macrae *et al.*, 2006 ▸) packing view, showing the C—H⋯π inter­actions (blue lines) building a three-dimensional network. H atoms are represented as small spheres of arbitrary radii.

The cohesion within the crystal is based on weak C—H⋯S and C—H⋯π inter­actions (Table 2 ▸). The C—H⋯S inter­actions build up a ribbon developing parallel to the (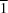
10) plane (Fig. 3 ▸). The C—H⋯π inter­actions link the ribbons to form a three-dimensional network (Fig. 4 ▸).

## Database survey   

A search in the Cambridge Structural Database (Version 5.36; Groom & Allen, 2014 ▸) reveals seven hits for related seven structures having a ferrocene moiety 1,2-disubstituted by a di­phenyl­thio­phosphoroyl and an allyl ether thiol (Mouas Toma *et al.*, 2014 ▸; Malacea *et al.*, 2013 ▸; Routaboul *et al.*, 2007 ▸).

## Synthesis and crystallization   

In a Schlenk tube, (**1**) (0.749 mg, 1.74 mmol) (see Fig. 1 ▸) was dissolved in dry di­chloro­methane (8 ml). A 54% solution of tetra­fluoro­boric acid in ether (0.73 ml, 5.30 mmol) was then added. After 1 min stirring, a solution of 4-hy­droxy­thio­phenol (20 mmol) in dry di­chloro­methane (8 ml) was added. After 1 min of stirring, the crude material was filtered on silica gel with ether as eluent. After evaporation of the solvent, (**2**) (0.73 g, 1.35 mmol) was obtained as a yellow solid (yield 78%). (**2**) (290 mg (5.38 × 10^−4^ *M*) and caesium carbonate (450 mg, 2.5 equivalents) in acetone (20 ml) were mixed for 2 min. Then, allyl bromide (0.047 ml, 1 equivalent) was added to the mixture, which was heated under reflux overnight. After cooling to room temperature, the product was recovered by chromatography on silica with petroleum ether/ethyl acetate (90/10). After evaporation of the solvent, compound (**3**) (yield 266 mg, 85%) was isolated as a yellow–orange powder.

## Refinement   

Crystal data, data collection and structure refinement details are summarized in Table 3 ▸. All H atoms were positioned geometrically and treated as riding on their parent atoms, with C—H = 0.95 (aromatic) or 0.99 Å (methyl­ene) and *U*_iso_(H) = 1.2*U*_eq_(C).

## Supplementary Material

Crystal structure: contains datablock(s) I, global. DOI: 10.1107/S2056989015013560/is5405sup1.cif

Structure factors: contains datablock(s) I. DOI: 10.1107/S2056989015013560/is5405Isup2.hkl

CCDC reference: 1412894

Additional supporting information:  crystallographic information; 3D view; checkCIF report

## Figures and Tables

**Table 1:** Comparison of geometrical parameters (, ) for the title compound and related structures Notes: ANG1 is the dihedral angle between the C2/C21/S2 plane and the Cp ring; S1-to-Cp1 and S2-to-Cp1 represent the perpendicular distance of the S atom to the substituted Cp ring plane; Cp1/Cp2 is the dihedral angle between the two Cp rings; C1P1, P1S1 and C2C21 are the bond lengths.

Refcode	ANG1	S1-to-Cp1	S2-to-Cp1	Cp1/Cp2	C1P1	P1S1	C2C21	C21-S2
This work	74.9(1)	1.263(5)	1.763(4)	3.94(15)	1.798(2)	1.9571(8)	1.499(3)	1.829(2)
CODXIE	89.5(1)	0.986(4)	1.751(3)	2.30(11)	1.792(2)	1.9572(6)	1.488(2)	1.835(2)
GIPPEC	73.1(4)	0.996(1)	1.748(2)	1.4(3)	1.788(4)	1.958(2)	1.496(5)	1.820(4)
GIPPEC	74.9(3)	1.155(1)	1.757(2)	2.4(3)	1.798(4)	1.956(2)	1.495(5)	1.817(4)
GIPPIG	15.8(2)	1.063(1)	0.457(1)	2.3(2)	1.792(2)	1.958(1)	1.500(3)	1.811(3)
GIPPOM	71.8(3)	0.921(1)	1.647(3)	1.5(2)	1.802(3)	1.957(1)	1.502(4)	1.825(3)
GIPPUS	73.9(6)	1.054(1)	1.638(3)	1.91(6)	1.789(8)	1.957(3)	1.502(11)	1.829(8)
GIPQAZ	77.1(2)	0.858	1.500(1)	0.70	1.788(2)	1.961(1)	1.491(3)	1.817(2)
LEXCOH	87.3(7)	0.83(2)	1.72(2)	2.0(4)	1.798(2)	1.957(8)	1.499(3)	1.829(2)

References for refcodes: CODXIE: Mouas Toma *et al.* (2014 ▸); GIPPEC, GIPPIG, GIPPUS and GIPQAZ: Malacea *et al.* (2013 ▸); LEXCOH: Routaboul *et al.* (2007 ▸).

**Table 2:** Hydrogen-bond geometry (, ) *Cg*1 and *Cg*2 are the centroids of the C111C116 and C6C10 rings, respectively

*D*H*A*	*D*H	H*A*	*D* *A*	*D*H*A*
C28H28*A*S2^i^	0.99	2.84	3.738(3)	150
C30H30*B*S1^ii^	0.95	2.83	3.663(3)	147
C126H126S1	0.95	2.85	3.341(2)	113
C4H4*Cg*1^iii^	0.95	2.81	3.63	146
C113H113*Cg*2^iv^	0.95	2.73	3.60	153

Symmetry codes: (i) 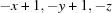; (ii) 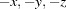; (iii) 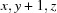; (iv) 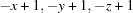.

**Table 3:** Experimental details

Crystal data	
Chemical formula	[Fe(C_5_H_5_)(C_27_H_24_OPS_2_)]
*M*_r_	580.49
Crystal system, space group	Triclinic, *P* 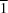
Temperature (K)	173
*a*, *b*, *c* ()	7.8161(3), 8.3179(3), 21.6998(6)
, , ()	97.773(3), 99.672(3), 95.329(3)
*V* (^3^)	1368.26(8)
*Z*	2
Radiation type	Mo *K*
(mm^1^)	0.79
Crystal size (mm)	0.55 0.50 0.07

Data collection	
Diffractometer	Agilent Xcalibur Eos (Gemini ultra)
Absorption correction	Multi-scan (*CrysAlis PRO*; Agilent, 2014 ▸)
*T*_min_, *T*_max_	0.637, 0.946
No. of measured, independent and observed [*I* > 2(*I*)] reflections	27439, 5592, 4965
*R*_int_	0.052
(sin /)_max_ (^1^)	0.625

Refinement	
*R*[*F* ^2^ > 2(*F* ^2^)], *wR*(*F* ^2^), *S*	0.044, 0.123, 1.10
No. of reflections	5592
No. of parameters	334
H-atom treatment	H-atom parameters constrained
_max_, _min_ (e ^3^)	1.06, 0.68

Computer programs: *CrysAlis PRO* (Agilent, 2014 ▸), *SIR97* (Altomare *et al.*, 1999 ▸), *SHELXL2014* (Sheldrick, 2015 ▸), *ORTEP-3 for Windows* (Farrugia, 2012 ▸), *Mercury* (Macrae *et al.*, 2006 ▸) and *PLATON* (Spek, 2009 ▸).

## supplementary crystallographic information

### Crystal data

**Table:**

[Fe(C_5_H_5_)(C_27_H_24_OPS_2_)]	*Z* = 2
*M**_r_* = 580.49	*F*(000) = 604
Triclinic, *P*1	*D*_x_ = 1.409 Mg m^−^^3^
*a* = 7.8161 (3) Å	Mo *K*α radiation, λ = 0.71073 Å
*b* = 8.3179 (3) Å	Cell parameters from 11224 reflections
*c* = 21.6998 (6) Å	θ = 3.5–29.3°
α = 97.773 (3)°	µ = 0.79 mm^−^^1^
β = 99.672 (3)°	*T* = 173 K
γ = 95.329 (3)°	Platelet, yellow
*V* = 1368.26 (8) Å^3^	0.55 × 0.50 × 0.07 mm

### Data collection

**Table:**

Agilent Xcalibur Eos (Gemini ultra) diffractometer	5592 independent reflections
Radiation source: Enhance (Mo) X-ray Source	4965 reflections with *I* > 2σ(*I*)
Graphite monochromator	*R*_int_ = 0.052
Detector resolution: 16.1978 pixels mm^-1^	θ_max_ = 26.4°, θ_min_ = 3.4°
ω scan	*h* = −9→9
Absorption correction: multi-scan (*CrysAlis PRO*; Agilent, 2014)	*k* = −10→10
*T*_min_ = 0.637, *T*_max_ = 0.946	*l* = −27→27
27439 measured reflections	

### Refinement

**Table:**

Refinement on *F*^2^	0 restraints
Least-squares matrix: full	Hydrogen site location: inferred from neighbouring sites
*R*[*F*^2^ > 2σ(*F*^2^)] = 0.044	H-atom parameters constrained
*wR*(*F*^2^) = 0.123	*w* = 1/[σ^2^(*F*_o_^2^) + (0.073*P*)^2^ + 0.8499*P*] where *P* = (*F*_o_^2^ + 2*F*_c_^2^)/3
*S* = 1.10	(Δ/σ)_max_ < 0.001
5592 reflections	Δρ_max_ = 1.06 e Å^−^^3^
334 parameters	Δρ_min_ = −0.68 e Å^−^^3^

### Special details

**Table:**

Geometry. All e.s.d.'s (except the e.s.d. in the dihedral angle between two l.s. planes) are estimated using the full covariance matrix. The cell e.s.d.'s are taken into account individually in the estimation of e.s.d.'s in distances, angles and torsion angles; correlations between e.s.d.'s in cell parameters are only used when they are defined by crystal symmetry. An approximate (isotropic) treatment of cell e.s.d.'s is used for estimating e.s.d.'s involving l.s. planes.

### Fractional atomic coordinates and isotropic or equivalent isotropic displacement parameters (Å^2^)

**Table:**

	*x*	*y*	*z*	*U*_iso_*/*U*_eq_	
Fe1	0.57578 (4)	0.69829 (4)	0.36521 (2)	0.01812 (12)	
S1	0.41109 (8)	0.22036 (7)	0.28912 (3)	0.02775 (16)	
S2	0.55111 (9)	0.62383 (10)	0.14588 (3)	0.03703 (18)	
P1	0.65260 (7)	0.31849 (7)	0.29668 (3)	0.01835 (14)	
O1	0.0549 (3)	0.2125 (2)	−0.06613 (8)	0.0341 (4)	
C1	0.6885 (3)	0.5381 (3)	0.30945 (10)	0.0180 (4)	
C2	0.5970 (3)	0.6457 (3)	0.27254 (10)	0.0209 (4)	
C3	0.6735 (3)	0.8083 (3)	0.29799 (11)	0.0246 (5)	
H3	0.6395	0.9045	0.2827	0.030*	
C4	0.8091 (3)	0.8031 (3)	0.34994 (11)	0.0245 (5)	
H4	0.8803	0.8950	0.3754	0.029*	
C5	0.8198 (3)	0.6372 (3)	0.35734 (10)	0.0199 (4)	
H5	0.8997	0.5985	0.3884	0.024*	
C6	0.5246 (3)	0.6660 (3)	0.45271 (11)	0.0268 (5)	
H6	0.5970	0.6220	0.4844	0.032*	
C7	0.3915 (3)	0.5764 (3)	0.40439 (12)	0.0283 (5)	
H7	0.3593	0.4616	0.3981	0.034*	
C8	0.3149 (3)	0.6875 (4)	0.36722 (12)	0.0331 (6)	
H8	0.2228	0.6603	0.3316	0.040*	
C9	0.4005 (4)	0.8474 (3)	0.39274 (13)	0.0330 (6)	
H9	0.3750	0.9457	0.3773	0.040*	
C10	0.5301 (3)	0.8339 (3)	0.44512 (12)	0.0288 (5)	
H10	0.6074	0.9215	0.4708	0.035*	
C21	0.4545 (3)	0.5985 (3)	0.21561 (11)	0.0254 (5)	
H21A	0.3599	0.6691	0.2183	0.030*	
H21B	0.4044	0.4835	0.2131	0.030*	
C22	0.3966 (3)	0.5028 (3)	0.08384 (11)	0.0289 (5)	
C23	0.3552 (4)	0.3363 (4)	0.08290 (12)	0.0356 (6)	
H23	0.4059	0.2859	0.1172	0.043*	
C24	0.2406 (4)	0.2436 (3)	0.03231 (13)	0.0362 (6)	
H24	0.2117	0.1302	0.0324	0.043*	
C25	0.1677 (3)	0.3150 (3)	−0.01840 (11)	0.0292 (5)	
C26	0.2098 (3)	0.4802 (3)	−0.01862 (12)	0.0308 (5)	
H26	0.1616	0.5296	−0.0536	0.037*	
C27	0.3232 (3)	0.5734 (3)	0.03275 (12)	0.0311 (5)	
H27	0.3508	0.6872	0.0329	0.037*	
C28	−0.0096 (3)	0.2804 (3)	−0.12162 (12)	0.0305 (5)	
H28A	0.0890	0.3363	−0.1369	0.037*	
H28B	−0.0896	0.3617	−0.1115	0.037*	
C29	−0.1045 (3)	0.1461 (3)	−0.17163 (13)	0.0332 (6)	
H29	−0.1928	0.0732	−0.1614	0.040*	
C30	−0.0706 (4)	0.1246 (4)	−0.22922 (13)	0.0409 (7)	
H30A	0.0172	0.1962	−0.2403	0.049*	
H30B	−0.1340	0.0376	−0.2598	0.049*	
C111	0.7998 (3)	0.2652 (3)	0.36315 (10)	0.0194 (4)	
C112	0.7401 (3)	0.2600 (3)	0.42011 (12)	0.0269 (5)	
H112	0.6228	0.2772	0.4225	0.032*	
C113	0.8504 (3)	0.2299 (3)	0.47306 (11)	0.0301 (5)	
H113	0.8095	0.2287	0.5118	0.036*	
C114	1.0212 (3)	0.2015 (3)	0.46948 (11)	0.0274 (5)	
H114	1.0971	0.1804	0.5058	0.033*	
C115	1.0806 (3)	0.2040 (3)	0.41296 (12)	0.0248 (5)	
H115	1.1970	0.1833	0.4105	0.030*	
C116	0.9712 (3)	0.2364 (3)	0.35992 (11)	0.0224 (5)	
H116	1.0131	0.2390	0.3214	0.027*	
C121	0.7425 (3)	0.2591 (3)	0.22639 (11)	0.0226 (5)	
C122	0.8897 (3)	0.3491 (3)	0.21468 (12)	0.0291 (5)	
H122	0.9439	0.4431	0.2437	0.035*	
C123	0.9575 (4)	0.3019 (3)	0.16083 (13)	0.0343 (6)	
H123	1.0593	0.3622	0.1533	0.041*	
C124	0.8764 (4)	0.1664 (3)	0.11795 (13)	0.0373 (6)	
H124	0.9217	0.1354	0.0807	0.045*	
C125	0.7303 (4)	0.0764 (3)	0.12911 (12)	0.0352 (6)	
H125	0.6756	−0.0165	0.0996	0.042*	
C126	0.6633 (3)	0.1212 (3)	0.18318 (11)	0.0267 (5)	
H126	0.5636	0.0583	0.1910	0.032*	

### Atomic displacement parameters (Å^2^)

**Table:**

	*U*^11^	*U*^22^	*U*^33^	*U*^12^	*U*^13^	*U*^23^
Fe1	0.02424 (19)	0.01838 (18)	0.01314 (18)	0.00476 (13)	0.00804 (13)	0.00005 (12)
S1	0.0243 (3)	0.0266 (3)	0.0302 (3)	−0.0024 (2)	0.0060 (2)	−0.0008 (2)
S2	0.0342 (4)	0.0605 (5)	0.0152 (3)	−0.0022 (3)	0.0066 (2)	0.0045 (3)
P1	0.0225 (3)	0.0173 (3)	0.0152 (3)	0.0031 (2)	0.0057 (2)	−0.0009 (2)
O1	0.0461 (11)	0.0333 (10)	0.0205 (9)	0.0090 (8)	−0.0001 (8)	−0.0002 (7)
C1	0.0229 (11)	0.0182 (10)	0.0136 (10)	0.0028 (8)	0.0074 (8)	−0.0009 (8)
C2	0.0277 (12)	0.0231 (11)	0.0144 (10)	0.0062 (9)	0.0097 (9)	0.0022 (8)
C3	0.0367 (13)	0.0212 (11)	0.0195 (11)	0.0053 (9)	0.0131 (10)	0.0045 (9)
C4	0.0300 (12)	0.0204 (11)	0.0233 (12)	−0.0012 (9)	0.0123 (9)	−0.0019 (9)
C5	0.0204 (11)	0.0233 (11)	0.0161 (11)	0.0015 (8)	0.0071 (8)	−0.0007 (8)
C6	0.0347 (13)	0.0340 (13)	0.0160 (11)	0.0074 (10)	0.0147 (10)	0.0048 (9)
C7	0.0321 (13)	0.0297 (12)	0.0261 (13)	0.0006 (10)	0.0191 (10)	0.0002 (10)
C8	0.0231 (12)	0.0560 (17)	0.0218 (12)	0.0081 (11)	0.0091 (10)	0.0023 (11)
C9	0.0404 (15)	0.0348 (13)	0.0326 (14)	0.0192 (11)	0.0217 (11)	0.0079 (11)
C10	0.0373 (14)	0.0295 (12)	0.0208 (12)	0.0047 (10)	0.0160 (10)	−0.0059 (9)
C21	0.0286 (12)	0.0332 (12)	0.0160 (11)	0.0075 (10)	0.0065 (9)	0.0035 (9)
C22	0.0294 (13)	0.0456 (15)	0.0139 (11)	0.0106 (11)	0.0092 (9)	0.0011 (10)
C23	0.0442 (16)	0.0457 (15)	0.0203 (13)	0.0223 (13)	0.0057 (11)	0.0063 (11)
C24	0.0514 (17)	0.0343 (14)	0.0244 (13)	0.0155 (12)	0.0067 (12)	0.0028 (10)
C25	0.0333 (13)	0.0388 (14)	0.0168 (12)	0.0133 (11)	0.0079 (10)	−0.0012 (10)
C26	0.0372 (14)	0.0387 (14)	0.0180 (12)	0.0107 (11)	0.0048 (10)	0.0057 (10)
C27	0.0364 (14)	0.0390 (14)	0.0186 (12)	0.0066 (11)	0.0068 (10)	0.0036 (10)
C28	0.0310 (13)	0.0389 (14)	0.0215 (12)	0.0085 (11)	0.0041 (10)	0.0025 (10)
C29	0.0272 (13)	0.0415 (15)	0.0292 (14)	0.0025 (11)	0.0022 (10)	0.0047 (11)
C30	0.0409 (16)	0.0482 (17)	0.0289 (15)	0.0022 (13)	0.0022 (12)	−0.0040 (12)
C111	0.0254 (11)	0.0156 (10)	0.0176 (11)	0.0034 (8)	0.0058 (8)	0.0010 (8)
C112	0.0258 (12)	0.0336 (13)	0.0237 (12)	0.0067 (10)	0.0103 (9)	0.0037 (10)
C113	0.0371 (14)	0.0387 (14)	0.0169 (12)	0.0065 (11)	0.0095 (10)	0.0052 (10)
C114	0.0319 (13)	0.0287 (12)	0.0197 (12)	0.0023 (10)	0.0007 (9)	0.0025 (9)
C115	0.0228 (11)	0.0248 (11)	0.0266 (12)	0.0040 (9)	0.0050 (9)	0.0019 (9)
C116	0.0273 (12)	0.0210 (10)	0.0204 (11)	0.0043 (9)	0.0096 (9)	0.0010 (8)
C121	0.0318 (12)	0.0211 (11)	0.0158 (11)	0.0088 (9)	0.0061 (9)	0.0006 (8)
C122	0.0399 (14)	0.0237 (11)	0.0259 (13)	0.0066 (10)	0.0128 (11)	0.0015 (9)
C123	0.0480 (16)	0.0325 (13)	0.0301 (14)	0.0117 (12)	0.0233 (12)	0.0071 (11)
C124	0.0564 (18)	0.0383 (14)	0.0234 (13)	0.0210 (13)	0.0177 (12)	0.0032 (11)
C125	0.0515 (17)	0.0307 (13)	0.0222 (13)	0.0144 (12)	0.0059 (11)	−0.0054 (10)
C126	0.0355 (13)	0.0234 (11)	0.0206 (12)	0.0074 (10)	0.0041 (10)	−0.0002 (9)

### Geometric parameters (Å, º)

**Table:**

Fe1—C2	2.037 (2)	C22—C23	1.389 (4)
Fe1—C1	2.038 (2)	C22—C27	1.389 (4)
Fe1—C3	2.038 (2)	C23—C24	1.384 (4)
Fe1—C10	2.040 (2)	C23—H23	0.9500
Fe1—C9	2.040 (2)	C24—C25	1.385 (4)
Fe1—C8	2.041 (2)	C24—H24	0.9500
Fe1—C4	2.045 (2)	C25—C26	1.384 (4)
Fe1—C5	2.048 (2)	C26—C27	1.391 (4)
Fe1—C7	2.049 (2)	C26—H26	0.9500
Fe1—C6	2.052 (2)	C27—H27	0.9500
S1—P1	1.9571 (8)	C28—C29	1.491 (4)
S2—C22	1.774 (3)	C28—H28A	0.9900
S2—C21	1.829 (2)	C28—H28B	0.9900
P1—C1	1.798 (2)	C29—C30	1.313 (4)
P1—C121	1.812 (2)	C29—H29	0.9500
P1—C111	1.820 (2)	C30—H30A	0.9500
O1—C25	1.376 (3)	C30—H30B	0.9500
O1—C28	1.434 (3)	C111—C116	1.394 (3)
C1—C5	1.437 (3)	C111—C112	1.397 (3)
C1—C2	1.440 (3)	C112—C113	1.382 (3)
C2—C3	1.425 (3)	C112—H112	0.9500
C2—C21	1.499 (3)	C113—C114	1.391 (4)
C3—C4	1.420 (4)	C113—H113	0.9500
C3—H3	0.9500	C114—C115	1.384 (3)
C4—C5	1.420 (3)	C114—H114	0.9500
C4—H4	0.9500	C115—C116	1.388 (3)
C5—H5	0.9500	C115—H115	0.9500
C6—C7	1.420 (4)	C116—H116	0.9500
C6—C10	1.425 (4)	C121—C122	1.393 (4)
C6—H6	0.9500	C121—C126	1.400 (3)
C7—C8	1.416 (4)	C122—C123	1.387 (3)
C7—H7	0.9500	C122—H122	0.9500
C8—C9	1.427 (4)	C123—C124	1.387 (4)
C8—H8	0.9500	C123—H123	0.9500
C9—C10	1.414 (4)	C124—C125	1.381 (4)
C9—H9	0.9500	C124—H124	0.9500
C10—H10	0.9500	C125—C126	1.383 (4)
C21—H21A	0.9900	C125—H125	0.9500
C21—H21B	0.9900	C126—H126	0.9500
			
C2—Fe1—C1	41.39 (9)	C6—C7—H7	125.8
C2—Fe1—C3	40.92 (9)	Fe1—C7—H7	126.4
C1—Fe1—C3	68.89 (9)	C7—C8—C9	107.9 (2)
C2—Fe1—C10	157.22 (10)	C7—C8—Fe1	70.06 (14)
C1—Fe1—C10	159.09 (10)	C9—C8—Fe1	69.50 (14)
C3—Fe1—C10	120.70 (10)	C7—C8—H8	126.1
C2—Fe1—C9	121.19 (10)	C9—C8—H8	126.1
C1—Fe1—C9	160.06 (11)	Fe1—C8—H8	126.0
C3—Fe1—C9	103.85 (10)	C10—C9—C8	107.9 (2)
C10—Fe1—C9	40.56 (11)	C10—C9—Fe1	69.70 (14)
C2—Fe1—C8	106.48 (10)	C8—C9—Fe1	69.57 (14)
C1—Fe1—C8	125.26 (10)	C10—C9—H9	126.0
C3—Fe1—C8	119.57 (11)	C8—C9—H9	126.0
C10—Fe1—C8	68.52 (11)	Fe1—C9—H9	126.3
C9—Fe1—C8	40.93 (12)	C9—C10—C6	108.2 (2)
C2—Fe1—C4	69.04 (9)	C9—C10—Fe1	69.74 (14)
C1—Fe1—C4	68.88 (9)	C6—C10—Fe1	70.08 (13)
C3—Fe1—C4	40.69 (10)	C9—C10—H10	125.9
C10—Fe1—C4	105.43 (10)	C6—C10—H10	125.9
C9—Fe1—C4	118.44 (10)	Fe1—C10—H10	125.9
C8—Fe1—C4	154.51 (11)	C2—C21—S2	107.47 (16)
C2—Fe1—C5	69.40 (9)	C2—C21—H21A	110.2
C1—Fe1—C5	41.19 (8)	S2—C21—H21A	110.2
C3—Fe1—C5	68.57 (9)	C2—C21—H21B	110.2
C10—Fe1—C5	121.60 (10)	S2—C21—H21B	110.2
C9—Fe1—C5	155.17 (11)	H21A—C21—H21B	108.5
C8—Fe1—C5	163.34 (11)	C23—C22—C27	118.8 (2)
C4—Fe1—C5	40.60 (9)	C23—C22—S2	121.7 (2)
C2—Fe1—C7	123.16 (10)	C27—C22—S2	119.4 (2)
C1—Fe1—C7	110.66 (9)	C24—C23—C22	120.4 (2)
C3—Fe1—C7	156.89 (11)	C24—C23—H23	119.8
C10—Fe1—C7	68.35 (10)	C22—C23—H23	119.8
C9—Fe1—C7	68.38 (11)	C23—C24—C25	120.5 (3)
C8—Fe1—C7	40.50 (11)	C23—C24—H24	119.8
C4—Fe1—C7	162.25 (10)	C25—C24—H24	119.8
C5—Fe1—C7	127.34 (10)	O1—C25—C26	124.2 (2)
C2—Fe1—C6	159.99 (10)	O1—C25—C24	115.9 (2)
C1—Fe1—C6	124.74 (9)	C26—C25—C24	119.8 (2)
C3—Fe1—C6	158.73 (10)	C25—C26—C27	119.5 (2)
C10—Fe1—C6	40.77 (10)	C25—C26—H26	120.3
C9—Fe1—C6	68.40 (10)	C27—C26—H26	120.3
C8—Fe1—C6	68.33 (10)	C22—C27—C26	121.0 (3)
C4—Fe1—C6	124.14 (10)	C22—C27—H27	119.5
C5—Fe1—C6	109.72 (10)	C26—C27—H27	119.5
C7—Fe1—C6	40.51 (10)	O1—C28—C29	108.9 (2)
C22—S2—C21	102.52 (12)	O1—C28—H28A	109.9
C1—P1—C121	104.21 (10)	C29—C28—H28A	109.9
C1—P1—C111	102.94 (10)	O1—C28—H28B	109.9
C121—P1—C111	106.95 (10)	C29—C28—H28B	109.9
C1—P1—S1	116.26 (8)	H28A—C28—H28B	108.3
C121—P1—S1	112.36 (8)	C30—C29—C28	122.5 (3)
C111—P1—S1	113.13 (8)	C30—C29—H29	118.8
C25—O1—C28	116.8 (2)	C28—C29—H29	118.8
C5—C1—C2	107.83 (19)	C29—C30—H30A	120.0
C5—C1—P1	125.49 (17)	C29—C30—H30B	120.0
C2—C1—P1	126.64 (17)	H30A—C30—H30B	120.0
C5—C1—Fe1	69.75 (12)	C116—C111—C112	119.2 (2)
C2—C1—Fe1	69.26 (12)	C116—C111—P1	122.39 (17)
P1—C1—Fe1	128.31 (11)	C112—C111—P1	118.36 (17)
C3—C2—C1	107.2 (2)	C113—C112—C111	120.5 (2)
C3—C2—C21	125.4 (2)	C113—C112—H112	119.7
C1—C2—C21	127.4 (2)	C111—C112—H112	119.7
C3—C2—Fe1	69.58 (13)	C112—C113—C114	119.9 (2)
C1—C2—Fe1	69.35 (12)	C112—C113—H113	120.0
C21—C2—Fe1	128.74 (16)	C114—C113—H113	120.0
C4—C3—C2	108.8 (2)	C115—C114—C113	119.9 (2)
C4—C3—Fe1	69.89 (13)	C115—C114—H114	120.0
C2—C3—Fe1	69.50 (13)	C113—C114—H114	120.0
C4—C3—H3	125.6	C114—C115—C116	120.4 (2)
C2—C3—H3	125.6	C114—C115—H115	119.8
Fe1—C3—H3	126.6	C116—C115—H115	119.8
C3—C4—C5	108.3 (2)	C115—C116—C111	120.0 (2)
C3—C4—Fe1	69.42 (13)	C115—C116—H116	120.0
C5—C4—Fe1	69.81 (13)	C111—C116—H116	120.0
C3—C4—H4	125.8	C122—C121—C126	119.4 (2)
C5—C4—H4	125.8	C122—C121—P1	120.81 (18)
Fe1—C4—H4	126.5	C126—C121—P1	119.81 (19)
C4—C5—C1	107.8 (2)	C123—C122—C121	120.2 (2)
C4—C5—Fe1	69.58 (13)	C123—C122—H122	119.9
C1—C5—Fe1	69.06 (12)	C121—C122—H122	119.9
C4—C5—H5	126.1	C122—C123—C124	119.9 (3)
C1—C5—H5	126.1	C122—C123—H123	120.1
Fe1—C5—H5	126.8	C124—C123—H123	120.1
C7—C6—C10	107.7 (2)	C125—C124—C123	120.4 (2)
C7—C6—Fe1	69.64 (13)	C125—C124—H124	119.8
C10—C6—Fe1	69.15 (13)	C123—C124—H124	119.8
C7—C6—H6	126.2	C124—C125—C126	120.1 (2)
C10—C6—H6	126.2	C124—C125—H125	119.9
Fe1—C6—H6	126.6	C126—C125—H125	119.9
C8—C7—C6	108.3 (2)	C125—C126—C121	120.0 (2)
C8—C7—Fe1	69.44 (14)	C125—C126—H126	120.0
C6—C7—Fe1	69.85 (13)	C121—C126—H126	120.0
C8—C7—H7	125.8		

### Hydrogen-bond geometry (Å, º)

*Cg*1 and *Cg*2 are the centroids of the C111—C116 and C6—C10 rings, respectively

**Table:**

*D*—H···*A*	*D*—H	H···*A*	*D*···*A*	*D*—H···*A*
C28—H28*A*···S2^i^	0.99	2.84	3.738 (3)	150
C30—H30*B*···S1^ii^	0.95	2.83	3.663 (3)	147
C126—H126···S1	0.95	2.85	3.341 (2)	113
C4—H4···*Cg*1^iii^	0.95	2.81	3.63	146
C113—H113···*Cg*2^iv^	0.95	2.73	3.60	153

Symmetry codes: (i) −*x*+1, −*y*+1, −*z*; (ii) −*x*, −*y*, −*z*; (iii) *x*, *y*+1, *z*; (iv) −*x*+1, −*y*+1, −*z*+1.
